# Drowning in a Water Bed

**Published:** 1860-07

**Authors:** 


*Drowning in a Water Bed.*—On Monday last an inquest was held at the County Lunatic Asylum, Colney Hatch, on the body of Martha Draper, one of the patients, she having been accidentally drowned in consequence of the bursting of a water bed.

